# Synchronization of Circadian Per2 Rhythms and HSF1-BMAL1:CLOCK Interaction in Mouse Fibroblasts after Short-Term Heat Shock Pulse

**DOI:** 10.1371/journal.pone.0024521

**Published:** 2011-09-07

**Authors:** Teruya Tamaru, Mitsuru Hattori, Kousuke Honda, Ivor Benjamin, Takeaki Ozawa, Ken Takamatsu

**Affiliations:** 1 Department of Physiology and Advanced Research Center for Medical Science, Toho University School of Medicine, Tokyo, Japan; 2 Department of Chemistry, School of Science, The University of Tokyo, Tokyo 133-0033, Japan; 3 University of Utah Health Science Center, Salt Lake City, Utah, United States of America; Vanderbilt University, United States of America

## Abstract

Circadian rhythms are the general physiological processes of adaptation to daily environmental changes, such as the temperature cycle. A change in temperature is a resetting cue for mammalian circadian oscillators, which are possibly regulated by the heat shock (HS) pathway. The HS response (HSR) is a universal process that provides protection against stressful conditions, which promote protein-denaturation. Heat shock factor 1 (HSF1) is essential for HSR. In the study presented here, we investigated whether a short-term HS pulse can reset circadian rhythms. Circadian *Per2* rhythm and HSF1-mediated gene expression were monitored by a real-time bioluminescence assay for *mPer2* promoter-driven luciferase and HS element (HSE; HSF1-binding site)-driven luciferase activity, respectively. By an optimal duration HS pulse (43°C for approximately 30 minutes), circadian *Per2* rhythm was observed in the whole mouse fibroblast culture, probably indicating the synchronization of the phases of each cell. This rhythm was preceded by an acute elevation in *mPer2* and HSF1-mediated gene expression. Mutations in the two predicted HSE sites adjacent (one of them proximally) to the E-box in the *mPer2* promoter dramatically abolished circadian *mPer2* rhythm. Circadian Per2 gene/protein expression was not observed in HSF1-deficient cells. These findings demonstrate that HSF1 is essential to the synchronization of circadian rhythms by the HS pulse. Importantly, the interaction between HSF1 and BMAL1:CLOCK heterodimer, a central circadian transcription factor, was observed after the HS pulse. These findings reveal that even a short-term HS pulse can reset circadian rhythms and cause the HSF1-BMAL1:CLOCK interaction, suggesting the pivotal role of crosstalk between the mammalian circadian and HSR systems.

## Introduction

Circadian clocks are endogenous oscillators that drive the daily rhythms of organisms ranging from bacteria to humans [1]. These clocks regulate various biochemical, physiological and behavioral processes with a periodicity of approximately 24 h. In almost all organisms studied to date, the core of the clock mechanism is a transcription/translation-based negative feedback loop, which relies on positive and negative oscillators [1], [2]. In vertebrates, three basic helix–loop–helix PAS (PER-ARNT-SIM) domain-containing transcription factors, called CLOCK and BMAL1, constitute the positive elements [3], [4]. CLOCK heterodimerizes with BMAL1 to form a transcriptionally active complex that binds to the E-box elements (CACGTG) present in the promoters of members of the *Period* (*Per*) and *Cryptochrome* (*Cry*) gene families. Once the PER and CRY proteins have been translated, they form heterodimers that can then translocate to the nucleus to repress BMAL1: CLOCK-mediated transcription through direct protein-protein interaction. These interactions then set up the rhythmic oscillations of *Per*, *Cry* and a large array of clock-controlled genes (CCGs) expression that drives the circadian clock-orchestrated physiological rhythms [5]. Under natural conditions, circadian rhythms are entrained to this 24 h day by environmental time cues, such as temperature and light [1]. Entrainment of the mammalian body temperature cycle was first described in mouse peripheral cells and tissues [6]. Even a slight change in temperature (36–38.5°C for approximately 6 h) acts as a resetting cue for the mammalian circadian oscillators, as monitored by the real-time circadian rhythm expression of *Per2* [7]. The heat shock (HS) pathway may be involved in this resetting process [7].

The HS response (HSR) is essential for protecting cells from protein-damaging stress associated with misfolded proteins and aging. Transient activation of heat shock factor 1 (HSF1) by diverse environmental and physiological stressors is essential for HSR. HSR involves a constitutive expression of the inactive HSF1 monomer, conversion to a DNA (HS element; HSE)-binding competent trimer, acutely enhanced transcription, and attenuation of HSF1-HSE binding [8]. HSF1 activates transcription of a large array of genes that encode proteins required for protein homeostasis, including molecular chaperones such as heat shock protein 70 and 90 (Hsp70 and Hsp90). These chaperones associate with HSF1 to inhibit HSF1 transcriptional activity [9]. However, HSF1 is not immediately released from its target promoter sites [10], which suggests that HSF1 remaining attached to the HSE sites transmits a signal to other pathways, such as those for CCGs expression. Indeed, previous reports have revealed crosstalk between the HSR and circadian systems [6], [7], [11]–[12], [13]. HSF1 is one of the transcription factors that exhibit circadian rhythmic chromatin binding [12]. Pharmacological inhibition of HSF1-mediated transcription impairs the entrainment of the circadian clock by the temperature cycle [7]. Furthermore, *Hsf1*-deficient (*Hsf1^−/−^*) mice [14] have a longer free-running period than wild-type littermates [12], which implies a combined role of HSF1 in the mammalian circadian and HSR systems.

In this study, we developed an easily applicable method to reset (synchronize) circadian rhythms by HS. We focused on the molecular basis for the synchronization of circadian rhythms by HS that mediates potential crosstalk between HSR and the circadian clock pathway. The results of our study suggest a molecular basis for thermotherapy and preventive medicine, including anti-cancer and anti-aging potential that is probably related to the circadian and HSR systems.

## Results and Discussion

### A short-term HS pulse resets circadian *mPer2* rhythms in NIH-3T3 fibroblasts

A circadian rhythm does not emerge in whole fibroblasts culture, since it is most likely that each individual cell has an endogenous rhythm with differential phases under a desynchronizing condition. Appropriate stimulation reveals the overt circadian rhythm in whole cultures by synchronizing (synchronously resetting) the phases of individual cells [15]–[17].

Several attempts have been made to reset (synchronize) circadian gene expression by exposing cells to HS temperatures, i.e., 42°C for 4–60 h [18] or 40°C for 150 min [11]. These reports revealed that among BMAL1:CLOCK-activated clock genes, *Per2* is a direct target for this synchronization. However, long-term exposure to high temperatures causes cell damage. Therefore, adequate short-term exposure may be preferable because it provides an easily applicable method to reset circadian rhythms by HS. To develop a better methodology for resetting circadian rhythms by HS, we optimized conditions for synchronization of circadian *mPer2* rhythms with a higher HS temperature (43°C) and a shorter duration.

**Figure 1:**
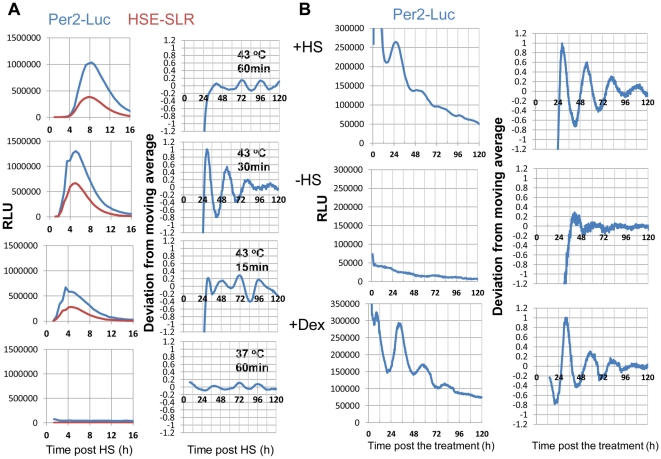
A short-term heat shock-pulse resets circadian *mPer2* rhythms in NIH-3T3 fibroblasts. (**A**) Determining the optimal duration for the heat shock (HS) treatment to induce circadian *mPer2* expression. NIH-3T3 fibroblasts stably expressing *mPer2* promoter-driven luciferase (*mPer2*-Luc) and 3×HSE-driven SLR luciferase (HSE-SLR) were treated with different durations of HS as indicated. Circadian *mPer2* (blue) and HS element (HSE) (red) profiles were monitored by a real-time dual-color bioluminescence assay. Relative acute (RLU) and normalized (deviation from moving average) circadian profiles from each experiment (average values from 5 independent experiments) are shown. (**B**) NIH-3T3 cells stably expressing *mPer2*-Luc were treated with or without an HS pulse (43°C, 30 min) or dexamethasone (Dex). The acute *mPer2*-Luc elevation pattern in the HS-stimulated cells is quite similar to the pattern shown in panel **A**.

We established NIH-3T3 fibroblasts, which stably express *mPer2* promoter-driven luciferase (Luc) and 3×HSE (containing three HSEs designed on the basis of HSE in the *hsp70* promoter and the TATA box)-driven SLR luciferase (HSE-SLR). Expression profiles of *mPer2* and HSE-transactivated genes were monitored by a real-time dual-color luciferase bioluminescence assay using the cells. In the cells, an acute *mPer2*-Luc and HSE-SLR elevation, followed by an overt circadian *mPer2*-Luc oscillation (period length; τ = 23.9±0.45) with an amplitude comparable to the dexamethasone (Dex)-synchronized rhythm (τ = 24.0±0.97), was observed after the optimal duration of the HS pulse (43°C, 30 min) (Fig. 1A, B). The HS pulse treatment may reset the circadian phases in each cells, and therefore render the circadian *mPer2*-Luc rhythm in the whole cell culture detectable. The circadian phase (peaks at approximately 30 and 54 h) detected by the HS pulse-synchronization was altered by approximately 3 h compared with the phase (peaks at approximately 33 and 57 h) detected by Dex-synchronization. This result is not necessarily inconsistent with that of a previous report, which showed that exposure to high temperatures causes a phase shift [7]. No acute and circadian *mPer2*-Luc and HSE-SLR peaks were detected in the absence of the HS pulse (Fig. 1A, B). A duration of 1 h resulted in wider acute *mPer2*-Luc and HSE-SLR peaks, followed by a much less obvious circadian *Per*2 oscillation. A shorter, 15-min duration resulted in smaller acute *mPer2*-Luc and HSE-SLR peaks, followed by a less overt circadian *Per*2 oscillation (Fig. 1A). These data suggest that early HS-elicited molecular events, possibly including HSF1-mediated transcription, may trigger synchronization of circadian *Per2* rhythms after an acute and adequate surge in *Per2* expression, which is achieved with an optimal HS pulse duration.

### An HSE identified proximal to the E-box is required for synchronization of circadian *Per2* rhythms by the HS pulse

**Figure 2:**
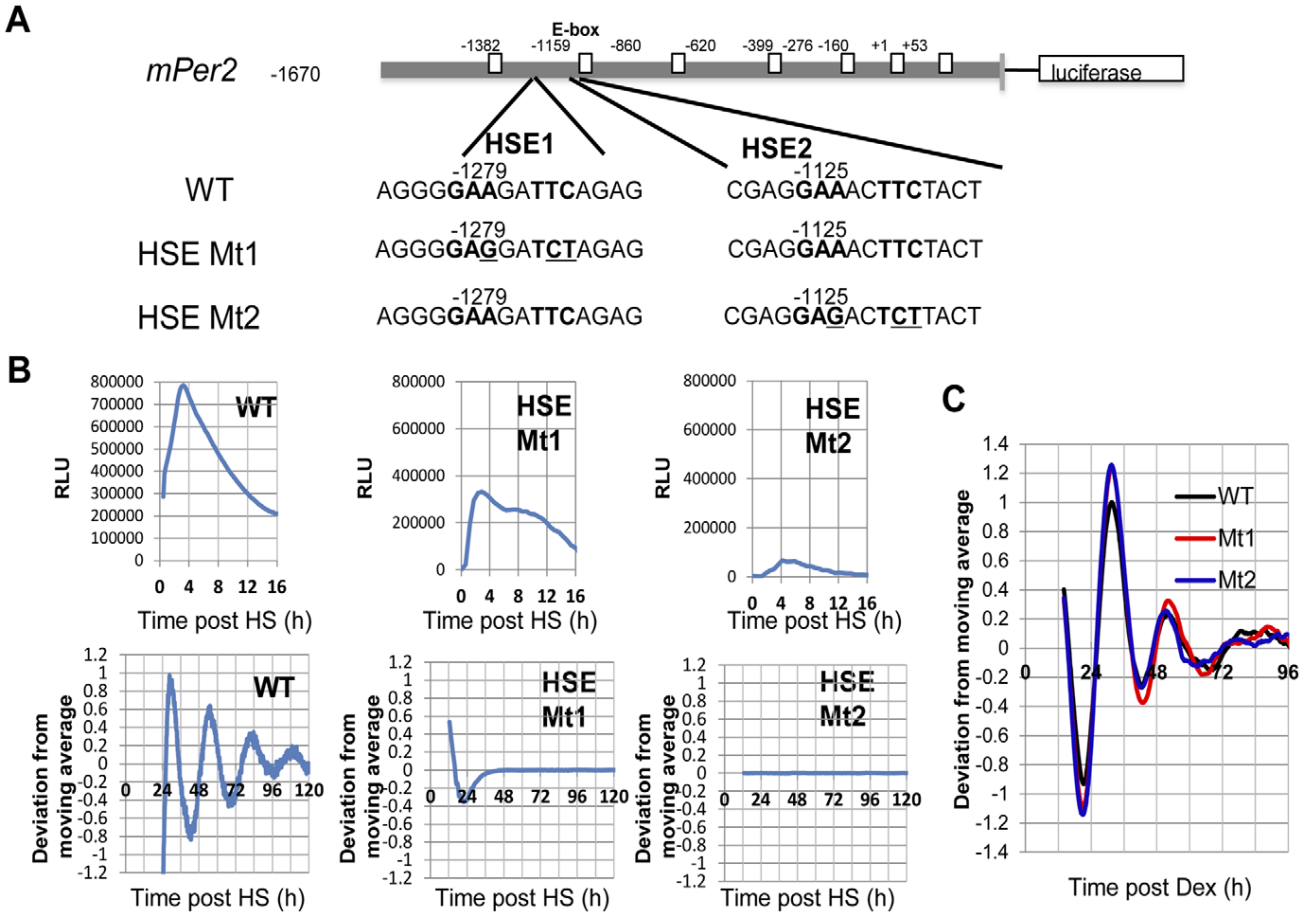
Determining the heat shock elements (HSEs) required for the heat shock (HS)-synchronized circadian *mPer2* rhythms. (**A**) Prediction of HSEs adjacent to the E-box elements (shown as quadrangles) in the *mPer2* promoter and a design for their mutagenesis (HSE Mt1 and Mt2) are shown. (**B, C**) NIH-3T3 cells transfected with *mPer2*-Luc and wild-type (WT) HSEs, HSE Mt1 and Mt2 were treated with the HS pulse or (**C**) dexamethasone (Dex). Circadian *mPer2* profiles were monitored by the real-time bioluminescence assay. Relative acute (RLU) and normalized (deviation from moving average) circadian profiles from each experiment (average values from 5 independent experiments) are shown.

To examine the structural basis of HSF1-mediated transcription that occurs during HS-synchronized circadian *Per*2 rhythms, we examined the effect of mutagenesis on the predicted HSEs in the *mPer2* promoter and on *mPer2*-Luc activity. Using a different methodology from that of a previous report merely that predicted HSEs [11], we searched HSF1-binding sites by sequence analysis of *mPer2* within the 1.7-kbp region located upstream of the transcription initiation site using TFSEARCH, and then checked the physiological effect by mutagenesis. We found only two potential HSF1-binding sites, HSEs (HSE1/2) with complete GAAXXTTC motifs (Fig. 2A, wild type [WT]). These sites were different from previously predicted sites: two complete TTCXXGAA motifs, one of them (centered around −1630) lying within a 22-bp sequence block [11]. Based on this sequence information, *mPer2* promoter-driven Luc constructs encoding the two mutants, HSE Mt1 (for HSE1) and Mt2 (for HSE2) (Fig. 2A) were expressed in NIH-3T3 cells. Compared with WT cells, Mt2-expressing cells exhibited dramatically decreased acute *mPer2*-Luc activity, followed by no *mPer2*-Luc rhythm after the HS pulse (Fig. 2B). Mt1-expressing cells exhibited significantly and comparatively, smaller decreases in acute *mPer2*-Luc activity, followed by no *mPer2*-Luc rhythm after the HS pulse (Fig. 2B). By Dex-synchronization, both Mt1 and Mt2-expressing cells exhibited a circadian *Per2* rhythm with patterns quite similar to that of the WT-expressing cells (Fig. 2C), which indicates that the impaired synchronization of the circadian *Per2* rhythms in Mt1- and Mt2- expressing cells was specific to the HS pulse. Notably, HSE2 was located at a highly proximal position (only approximately 30 bp) to the nearest E-box, whereas HSE1 was located farther (approximately 100 bp) from the nearest E-box. This proximity between HSE and the E-box may provide a plausible reason why an Mt2 mutation results in a more dramatic impairment of acute and circadian *mPer2* expression than an Mt1 mutation. According to a previous report [9], HSF1 is not immediately released from HSEs after the HS pulse. Hence, it is possible that, the high proximity between HSE2 and the E-box in *mPer2* promoter results in increased accessibility between HSF1 (remains to bind HSE2) and BMAL1:CLOCK (capable of binding to the E-box) after the HS pulse. We hypothesize that this potential accessibility between HSF1 and BMAL1:CLOCK may confer the capability to transmit the signal from the system for acute HS-induced gene expression to the system for synchronization of *Per2* rhythms. These data reveal that HSE2 and HSE1 are the primary and secondary sites, respectively, in the *mPer2* promoter required for the HS-synchronized circadian *mPer2* rhythms.

### HSF1 is essential to reset circadian Per2 rhythms by the HS pulse

**Figure 3:**
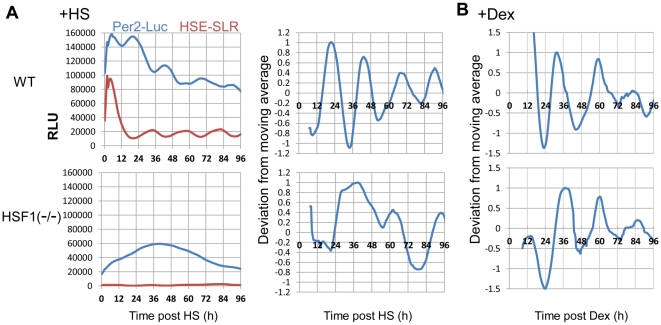
Heat shock factor 1 (HSF1) deficiency abolishes the heat shock (HS)-synchronized circadian *mPer2* rhythms and the heat shock element (HSE)-driven acute induction. Wild-type (WT) and *Hsf1* ^−/−^ mouse embryonic fibroblasts (MEFs) transfected with *mPer2*-Luc and HSE-SLR were treated with the (**A**) HS pulse or (**B**) dexamethasone (Dex). Circadian profiles were monitored by the real-time bioluminescence assay. Relative acute or normalized (deviation from moving average) profiles from each experiment (average values from 5 independent experiments) are shown.

To verify the role of HSF1-mediated transcription in HS-synchronized circadian *Per*2 rhythms, we examined the effect of HSF1 deficiency on detecting *mPer2*-Luc and PER2 rhythms after the HS pulse. In WT (*Hsf1^+/+^*) mouse embryonic fibroblasts (MEFs), an overt circadian rhythm of *mPer2*-Luc activity (τ = 23.5±0.65) preceded by an acute elevation of *mPer2*-Luc and HSE-SLR activity were observed after the HS pulse (Fig. 3A). The period length was quite similar to that of the NIH-3T3 cells, although differences were observed between MEFs and NIH-3T3 cells in the circadian phase (12±2.1 h) and acute *Per2* elevation pattern (the surge in *Per2* expression was smaller in MEFs). In contrast, after the HS pulse, neither an obvious circadian *mPer2*-Luc rhythm nor an acute elevation in HSE-SLR activity was observed in the *Hsf^−/−^* MEFs derived from *Hsf1^−/−^* mice (Fig. 3A) [14]. However, by Dex-synchronization, *Hsf^−/−^* MEFs exhibited a circadian *Per2* rhythm with a longer circadian period (τ = 23.8±0.60), as previously reported in *Hsf^−/−^* mice [12], than WT MEFs (τ = 22.8±0.54). These results indicate that the impaired synchronization of the circadian *Per2* rhythms in *Hsf^−/−^* MEFs was specific to the HS pulse. Similar to NIH-3T3 cells, WT MEFs exhibited an altered (approximately 10 h) circadian phase (peak at approximately 21 h) after the HS pulse compared with the phase (peak at approximately 31 h) after Dex treatment.

**Figure 4:**
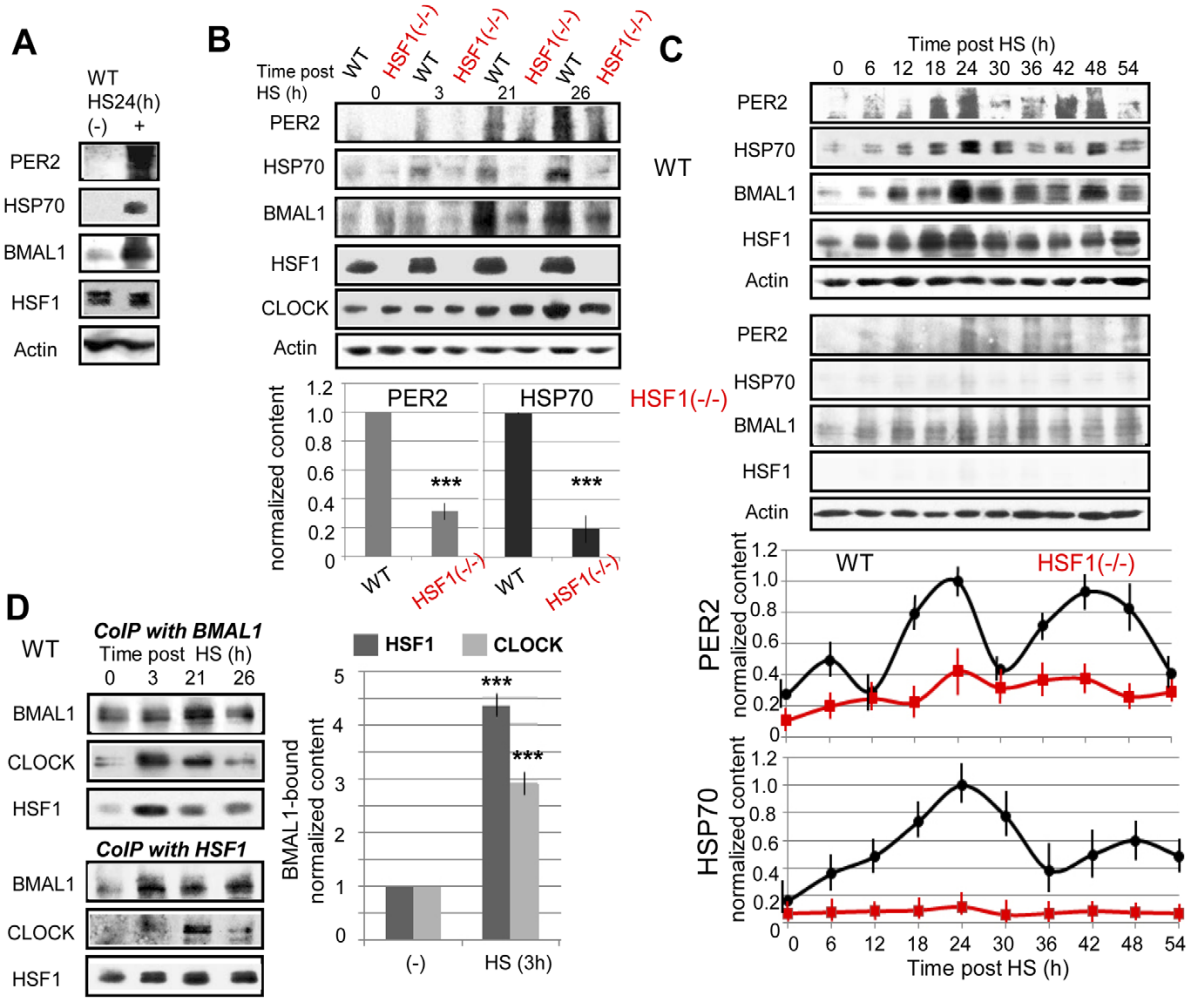
Heat shock factor 1 (HSF1) interacts with BMAL1:CLOCK after the heat shock (HS) pulse. Wild-type (WT) mouse embryonic fibroblasts (MEFs) were treated with or without an HS pulse. At 24 h after the HS pulse, the lysates were subjected to immunoblotting for PER2, HSP70, BMAL1, HSF1, and actin. Representative images from triplicate independent experiments are shown (**A**). WT and *Hsf1* ^−/−^ MEFs were treated with the HS pulse. At the indicated times after the HS pulse, the lysates (**B**) and BMAL1/HSF1 immunoprecipitates (**D; CoIP**) were subjected to immunoblotting for PER2, HSP70, BMAL1, HSF1, CLOCK, and actin. Representative images from triplicate independent experiments are shown. Normalized PER2 and HSP70 protein levels (**B**; at 26 h after the HS pulse), and BMAL1 coimmunoprecipitated with HSF1 and CLOCK (**D**) are shown as average values from triplicate independent experiments. Error bars indicate standard deviation (SD) (*** P<0.001). (**C**) WT and *Hsf1* ^−/−^ MEFs were treated with the HS pulse. At the indicated times after the HS pulse, the lysates were subjected to immunoblotting for PER2, HSP70, BMAL1, HSF1, and actin. Representative images from triplicate independent experiments are shown. Normalized circadian PER2 and HSP70 protein profiles plotted with average values from triplicate independent experiments are shown and error bars indicate SD.

To confirm the above results at the protein level, we examined protein expression patterns by immunoblotting after the HS pulse. In WT MEFs, circadianPER2 and BMAL1 expression as well as elevated HSP70 (a typical HS-induced protein) expression was observed after the HS pulse (Fig. 4A, B, D). PER2 and HSP70 levels after the HS pulse were significantly higher in WT MEFs than those in *Hsf1^−/−^* MEFs (Fig. 4 B, D). Circadian PER2 and BMAL1 expression was observed after the HS pulse over 2 days in WT MEFs but not in *Hsf1^−/−^* MEFs (Fig. 4B and D). Taken together, these results revealed that HSF1-dependent transcriptional regulation is essential to reset the circadian rhythms after the HS pulse.

### HSF1 interacts with BMAL1:CLOCK after the HS pulse

To test the theory that an acute interaction between HSF1 and BMAL1:CLOCK, which may occur adjacent to the E-box proximal to HSE, is involved in HS-synchronized circadian rhythms, we examined the association between HSF1 and BMAL1:CLOCK by co-immunoprecipitation.

The BMAL1 immunoprecipitate from WT MEFs at 3 and 21 h after the HS pulse, which matched the timing of the *Per2* elevation, contained significantly higher levels of HSF1 and CLOCK than those before or without the HS pulse (Fig. 4C). The HSF1 immunoprecipitate from WT MEFs after the HS pulse contained higher levels of BMAL1 and CLOCK than those before the HS pulse (Fig. 4C). An acute elevation in the HSF1-BMAL1:CLOCK interaction was more clearly observed in the co-immunoprecipitate with the anti-BMAL1 antibody than with the anti-HSF1 antibody, which demonstrates that the HSF1 content in the BMAL1:CLOCK complex shares a larger proportion than the BMAL1:CLOCK content in the HSF1 complex. By immunoprecitipation after the HS pulse, we detected a complex formation between HSF1 and BMAL1:CLOCK probably with two potential mechanisms. First, this formation may represent a direct binding between the two transcriptional activators (HSF1 and BMAL1, or HSF1 and CLOCK), and one may modify the activities of the other. Alternatively, this interaction may be DNA-mediated due to the proximity of these factors' binding sites (HSE and E-box) to each other. The acute and circadian recruitment of HSF1 to the BMAL1:CLOCK complex via these potential mechanisms after the HS pulse possibly mediates the synchronization of the circadian rhythms.

In summary, these investigation using mouse fibroblasts led to the following conclusions: (I) a short-term HS pulse (43°C, 30 min) may be sufficient to reset circadian *Per2* rhythms, (II) an acute surge in *Per2* expression probably under the control of HSF1-mediated transcription after the HS pulse, precedes the emergence of circadian *Per2* rhythm; (III) among the two adjacent HSE sites, HSE2, proximal to the E-box in the *mPer2* promoter, is required for the acute surge in *Per2* expression and following the emergence of the circadian *Per2* rhythm, which is possibly achieved by the HSF1-BMAL1:CLOCK interaction; and (IV) HSF1 is essential to reset circadian rhythms after the HS pulse. Furthermore, the identified HSE sites and HSF1-BMAL1:CLOCK interaction on the *Per2* promoter are potentially involved in the temperature resetting process through a small temperature change such as the circadian change in body temperature [6], [7], which is probably controlled by HSF1-mediated transcription, in the mammalian circadian system. We believe that our findings provide a clue to elucidating the physiological roles of the crosstalk between HSR and circadian systems, and thereby contribute to the identification of novel therapeutic targets for diseases such as cancer and metabolic syndrome implicated by these systems [19]–[22].

The experimental paradigm using a short-term HS pulse (43°C, 30 min) probably relates to the optimal conditions for the cooperative functioning of circadian and HSR system in the fibroblasts and potentially for other cells in the body. This paradigm probably relates to suitable conditions for thermotherapy (hyperthermia therapy) [23]–[25], as compared to other paradigms [7], [11], [15] with longer durations, which increase the unfavorable aspects of the HS-stimulus. Using the HS-pulse treatment, dysfunctional (or not completely functional) tissues and organs as a result of the irregular desynchronization of cellular clocks, caused by an irregular daily life style, aging, or tissue injury [26]–[28], are expected to be recovered effectively via resynchronization of cellular clocks. This resynchronization may help to achieve circadian-controlled regular coupling of physiological functions, such as circadian- controlled cell division, metabolism and stress-protection. Our findings offer a cellular/molecular basis for the development of thermotherapy and preventive medicine including anti-cancer and anti-aging.

## Materials and Methods

### Plasmid construction

The pGL4-Per2-Bsd vector was constructed to express the 1.7-kbp *mPer2* promoter-driven destabilized luciferase reporter, as described previously [29]. The pSLR- 3×HSE-Hyg vector was constructed by ligating the below mentioned synthetic DNA fragment into the MluI-BgIII site upstream of the destabilized SLR luciferase in pSLR (PEST)-test (Toyobo, Japan).

3×HSE: 5′-ACGCGTCTGAACGTTCTAGAACGTAGGGTCATACTGCTAGAACGTTCTAGAA CGTCTAGAACGTTCTAGAACGACTAGAGGGTATATAAAGGAAGCTCGACTTCCAGAGATCT-3′ ( GAAXXTTC ; HSE motif) HSF1-binding sites (HSE1/2) with a complete GAAXXTTC motif were found by sequence analysis of *mPer2* within the 1.7-kbp region located upstream of the transcription initiation site using TFSEARCH (http://www.cbrc.jp/research/db/TFSEARCH.html) (Fig. 2A, WT). pGL4-Per2 vectors with the two mutants HSE Mt1 (for HSE1) and Mt2 (for HSE2) were generated by exchanging nucleotides using the QuikChange site-directed mutagenesis kit (Fig.2A; Stratagene, USA).

### Cell culture and transfection

Mouse NIH-3T3 fibroblasts (RIKEN cell bank, Japan) and MEFs [14] were cultured as described previously [29], [30]. For the synchronization of circadian clock gene/protein expression, cells were cultured to confluence in DMEM with 10% fetal bovine serum at 37°C, treated with an HS pulse (optimal condition: 43°C, 30 min) and 0.1 µM Dex for 2 h. The medium was then changed to DMEM with 10% fetal bovine serum (50 mM Hepes, pH7.4 and 0.1 mM Luciferin (TOYOBO, Japan) were also add in the real-time bioluminescence assay), and the cells were cultured at 37°C.

DNA transfection was performed using Fugene HD (Roche Applied Science, Japan) according to the manufacturer's protocol.

Clones of NIH-3T3 cells stably expressing pGL4-Per2-Bsd and pSLR-3×HSE-Hyg were obtained by picking up the colony after treatment with 10 µg/ml blasticidin S (Invitrogen, Japan) and 200 µg/ml hygromycin B (Roche Applied Science) for 2–4weeks, respectively, according to the manufacturer's protocol.

### Real-time bioluminescence assay and data processing

Cells stably expressing the above-mentioned reporter vectors or transiently transfected with the plasmids were synchronized with 0.1 µM Dex, as described previously [29], [30] or an HS pulse (optimal condition: 43°C, 30 min). Real-time bioluminescence activities were monitored with 0.1 mM luciferin in the medium using Kronos (ATTO, Japan), as described previously [29]. The dual-color luciferase bioluminescence assay was performed using a spectroscopic filter attached to Kronos according to the manufacturer's protocol.

The values were obtained from each sample using the same detectors in the same experiments. If the y-axis is shown as a “deviation from the moving average,” the values were detrended according to the manufacturer's protocol (Kronos; ATTO, Japan). All values were normalized using maximum circadian peak intensities over time, except for the values obtained for the acute peak. The normalized data were further normalized by averaging intensity over time. Period length was deduced from each processed datum without averaging the data from each detector (the number of independent experiments is described in the figure legend), as described above, and shown as value±standard deviation (SD), which was calculated using Excel (Microsoft, Japan). The amplitude and phase was deduced from each processed datum with averaging the data from multiple detectors (the number of independent experiments is described in each figure legend).

### Biochemical analyses

Immunoprecipitation and immunoblotting were performed as described previously [29]–[31], using anti-BMAL1 [30], CLOCK (Affinity Bioreagents, USA), PER2 (Alpha Diagnostic Intl. USA), HSP70 (Upstate Biotechnology, USA), HSF1 (Upstate Biotechnology), and actin (loading control; Santa Cruz Biotechnology, USA) antibodies as well as HRP-conjugated anti-rabbit/goat/mouse IgG (Zymed, USA). Immunoblotting data were quantified by computerized densitometry, as described previously [31].

The immunoblotting values, which were simultaneously obtained from each independent experiment, were normalized by the loading control values (Fig. 4B, D). The normalized values were further normalized using the values of WT samples (Fig. 4B), maximum peak intensities over time (Fig. 4D), or values of the control (Fig. 4C). SDs of the values from multiple independent experiments (as described in each figure legend) were calculated using Excel. SDs are shown as error bars.

### Statistical analyses

We used factorial design analysis of τ-tests to analyze the data and to calculate p-values by Excel (Fig. 4), as appropriate. Data presented in this study represent the average of many experiments, all of which generated highly reproducible and consistent results.
